# Clinical applications of liquid biopsy as prognostic and predictive biomarkers in hepatocellular carcinoma: circulating tumor cells and circulating tumor DNA

**DOI:** 10.1186/s13046-018-0893-1

**Published:** 2018-09-03

**Authors:** Jie Li, Xu Han, Xiaona Yu, Zongzhen Xu, Guangsheng Yang, Bingqi Liu, Peng Xiu

**Affiliations:** 1 Department of General Surgery, Shandong Provincial Qianfoshan Hospital, Shandong University, Jinan, 250014 Shandong China; 2 Department of Hepatobiliary Surgery, Zibo Central Hospital, Zibo, 255000 Shandong China; 3 Department of General Medicine, Weifang Rongfu Military Hospital, Weifang, 261000 Shandong China

**Keywords:** Hepatocellular carcinoma, Liquid biopsy, Circulating tumor cells, Circulating tumor DNA

## Abstract

Hepatocellular carcinoma (HCC) is a highly malignant disease with a poor prognosis and high mortality due to a low early diagnosis rate, resistance to systemic treatments and progression to late-stage liver disease. Owing to limitations in the detection of HCC and the lack of awareness of healthcare systems, fewer than 40% of HCC patients are eligible for surgery due to advanced stages of the disease at the time of diagnosis and the occurrence of multiple lesions in the cirrhotic or fibrotic liver. At present, the updated American Association for the Study of Liver Disease (AASLD) guidelines no longer recommend alpha-fetoprotein (AFP) testing as a part of diagnostic evaluation. Thus, it is imperative to establish a novel diagnostic strategy with high sensitivity and reliability to monitor risk factors to detect HCC at an early stage. In recent years, “liquid biopsy,” (including circulating tumor cells (CTCs) and circulating tumor DNA (ctDNA)), has emerged as a technique for the characterization of circulating cells, providing a strong basis for the individualized treatment of patients. As a noninvasive detection method, liquid biopsy is expected to play an important role in the early diagnosis, dynamic monitoring of cancer patients and drug screening. In this review, we will focus on the clinical applications, recent studies and future prospects of liquid biopsy, particularly focusing on HCC.

## Background

Hepatocellular carcinoma (HCC) is the fifth most common cancer worldwide and remains the third most frequent cause of cancer death, with nearly 321,200 deaths and 366,100 new cases reported in China [1, 2]. The risk factors for the development of HCC include liver cirrhosis resulting from viral infections caused by hepatitis B virus (HBV) and- /or hepatitis C virus (HCV), excessive alcohol intake, Wilson’s disease, stage IV primary biliary cirrhosis and environmental exposure to aflatoxins [3, 4]. Although primary prevention of HBV infection through vaccination in infants has been shown to be effective in children in China and liver cancer deaths were reduced by 95% in the younger population (ages 0–19 years) 15 years after the implementation of an HBV vaccination program in high-risk areas in China in 1986, it may be too early for the incidence trend to be affected across all age groups [5].

Despite modern management, including the introduction of improved surgical techniques, comprehensive treatment and targeted therapies, the overall survival (OS) rates of HCC patients have not significantly improved. In addition, HCC is relatively chemotherapy resistant, and surgical interventions including partial liver resection and liver transplantation remain the only realistic treatment options for HCC. However, owing to the limitations in detection and lack of awareness of healthcare systems, fewer than 40% of HCC patients are eligible for surgery due to advanced stages of the disease at diagnosis and the occurrence of multiple lesions in the cirrhotic or fibrotic liver [6]. Various efforts have been made to improve survival rates through early screening methods based on serum alpha-fetoprotein (AFP) and liver ultrasound, which are the most widely used methods for HCC screening; however, with a sensitivity of 25% to 65% for AFP and 60% for ultrasound, the detection of a disease with such a high impact through these methods is remains suboptimal [7]. Therefore, it is imperative that diagnostic methods be improved to detect HCC at an early stage so that effective treatment can be administered in patients with HCC and metastatic colorectal cancer.

Although serum AFP has long been used as a marker for HCC screening and surveillance, it is not a sensitive or specific diagnostic marker for HCC. Furthermore, AFP levels can be elevated in non-HCC diseases, including chronic liver ailments, such as cirrhosis and hepatic inflammation; intrahepatic cholangiocarcinoma; and metastatic colon cancer [8]. Although serum AFP levels are efficient at predicting disease outcomes and monitoring tumor progression in AFP-producing HCC patients, the updated American Association for the Study of Liver Disease (AASLD) guidelines no longer recommend AFP testing as a part of diagnostic evaluation [9]. In these guidelines, the assessment of diameters of hepatic nodules, computed tomography (CT), magnetic resonance imaging (MRI) or tissue biopsy are recommended for the diagnosis of HCC.

Currently, the detection of the molecular drivers of tumors and of specific DNA mutations in tumor biopsy samples has become routine clinical practice in the era of individualized medicine, with the purpose of evaluating specific biomarkers to predict response or resistance to targeted agents. However, owing to tumor heterogeneity, described by different genomic profiles in both “space and time” in anatomically different areas of the same primary tumor and in metastases, it might not be sufficient to characterize the genetic heterogeneity of tumor with a single biopsy [10, 11]. Moreover, acquired drug resistance to targeted agents is common during the course of disease. These findings indicate an urgent need for the identification of additional cancer-specific biomarkers for early diagnosis, tumor evolution monitoring and prognostic prediction.

**Fig. 1:**
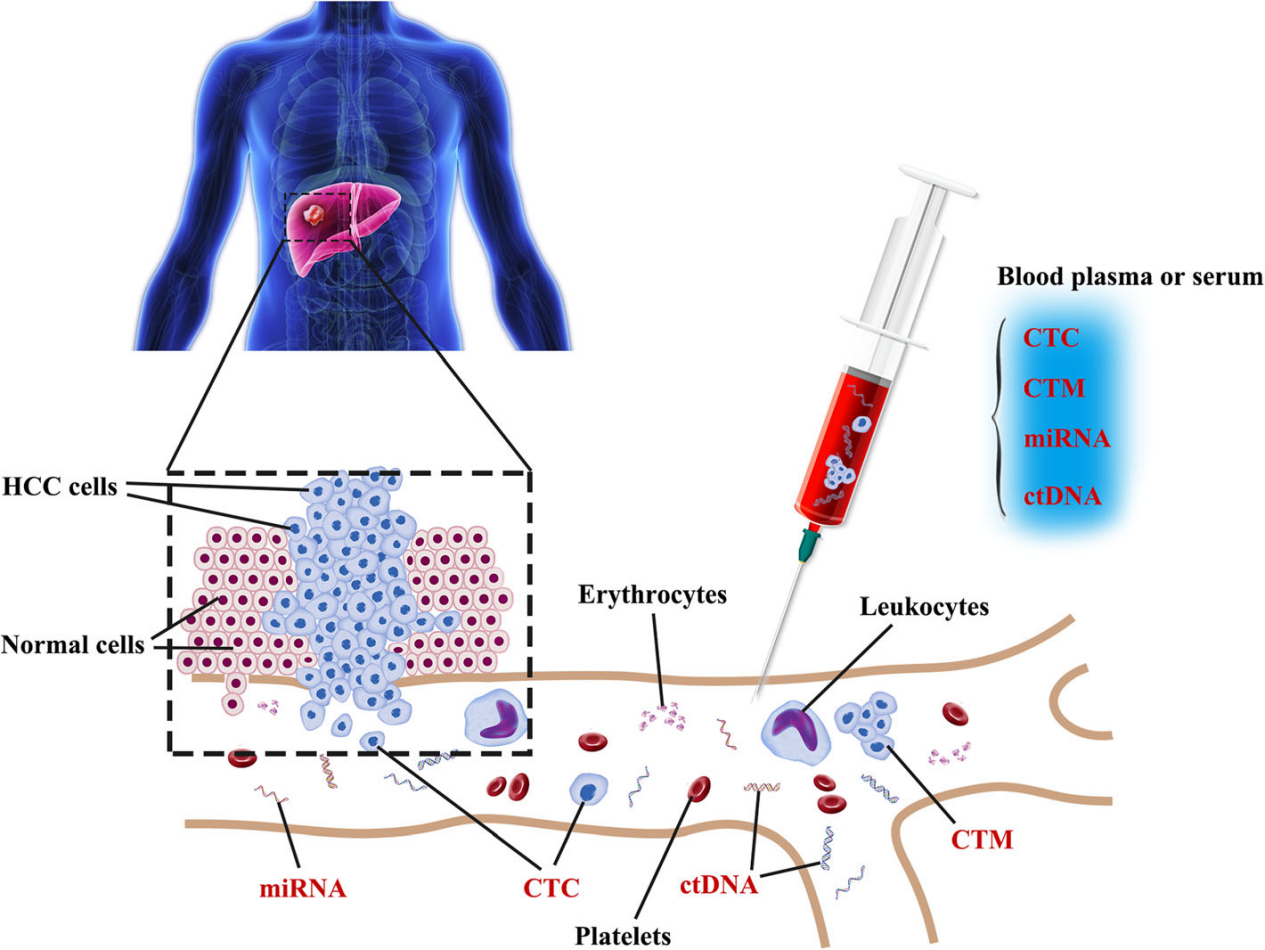
Liquid biopsy of HCC: circulating tumor cells (CTCs) and circulating tumor DNA (ctDNA) are easily accessible in peripheral blood of patients. These molecules are released from HCC cells undergoing apoptosis or necrosis and can be extracted from a blood sample. Analysis of these molecules can be used for early tumor detection and provide prognostic treatment strategy from HCC patients

Unlike tissue biopsy, liquid biopsy (including circulating tumor cells (CTCs) and circulating tumor DNA (ctDNA)) is based on obtaining a sample in a convenient and minimally invasive manner at multiple time points over the course of disease. Liquid biopsy enables the noninvasive detection and characterization of cancer, prediction of treatment response, monitoring of disease relapse and identification of mechanisms of resistance to targeted therapies. In this article, we will focus on clinical applications, recent studies and future prospects of liquid biopsy, particularly focusing on HCC (Fig. 1).

### Biology, Detection and enrichment of CTCS

CTCs were first discovered by the Australian doctor Thomas R. Ashworth in 1869 in the blood of a breast cancer patient [12]. CTCs are tumor cells that are transferred from a primary solid tumor to the peripheral circulation or lymphatic system to be circulated and eventually grow in the blood, bone marrow, lymph nodes or other healthy organs [13]. This process occurs at every stage of tumor development. In other words, CTCs are useful markers for early diagnosis and monitoring of disease relapse. However, studies of CTCs have been hampered for decades because these cells are present at extremely low frequencies in patient blood. During the metastatic process, these cells must struggle to survive in the bloodstream and less than 0.01% of CTCs introduced into the circulation survive to produce metastases [14]. Therefore, CTC detection has become a bottleneck, and improving the detection process has proven difficult.

In recent years, with improvements in technology, the separation and enrichment of CTC have been greatly improved. These separation and enrichment methods can be classified into two types based on the physical properties or biological properties of CTCs.

Physical methods mainly depend on the physical properties of CTCs, including size, density, malleabiliby, migratory capacity and electric charge. The basic method of size-based enrichment of CTCs, such as isolation by the size of epithelial tumor cells (ISET), is used to isolate epithelial tumor cells based on the assumption that tumor cells (~ 17–52 μm) are relatively larger than red blood cells (RBCs) (~ 6–8 μm) and white blood cells (WBCs) (~ 7–15 μm) [15]. Gradient centrifugation is another method that can select for CTCs by centrifugation on a Ficoll density gradient based on differences in density between tumor cells and blood cells [16]. Due to the substantial difference among tumor cells in a patient’s tumor or among different patients, some blood cells may display similar physical properties to CTCs; therefore, these physical methods have a higher false-positive rate, which limits their use.

Biological property-based technologies depend on antigen-antibody binding and specific antibodies that bind to surface markers on CTCs, including epithelial cell adhesion molecule (EpCAM), human epidermal growth factor receptor (Her2), members of the cytokeratin (CK) family (CK8, CK18 and CK19) and mesenchymal markers (N-cadherin and vimentin) [17–19]. The principle of the immune capture method is to target a specific antigen; this is accomplished by magnetic beads conjugated to a corresponding antibody, which is then used to bind to target cells to create a “target cell-antigen-antibody-magnetic bead” complex under the action of a magnetic field in a certain direction to enrich for target cells. There are two methods of immune capture: positive enrichment and negative enrichment. Positive enrichment uses a combination of magnetic beads bound to anti-target cell antibodies to separate tumor cells directly under the influence of magnetic field. The most representative positive enrichment method is the Cell-Search™ System (CSS: Veridex LLC, NJ, USA), which is the first and only product in the world that has been approved by the US Food and Drug Administration (FDA) and the Chinese National Food and Drug Administration (CFDA) for the detection of CTCs for the diagnosis of malignant diseases. In this platform, anti-EpCAM antibody-coated ferromagnetic beads are used at the initial step to enrich for CTCs, after which CK, CD45 and DAPI staining are used to confirm the presence of CTCs and remove leukocytes [20]. In the early market for CTC technologies, this system was considered to have good repeatability, sensitivity and specificity (it only requires 7.5 ml of blood, for the detection of CTCs among a number of blood cells and WBCs). However, it is not able to capture CTCs that may have lost these specific molecules, such as EpCAM, during epithelial-mesenchymal transition (EMT). Furthermore, the expression of tumor cell surface molecules, such as EpCAM on many solid epithelial tumors, is very heterogeneous or even undetectable (such as in the case of melanoma), which results in insufficiency and limitations, and even restrict the clinical application of EpCAM-dependent strategies for directly capturing CTCs. Moreover, following antibody cross-linking of cell surface antigens, CTCs captured by anti-EpCAM antibody no longer remain as unstimulated naive cells, and this leads to intracellular instability of the isolated CTCs, making them unsuitable for subsequent protein, molecular and genomic analyses. These limitations led to the development of the negative enrichment method. The Cytelligen system, considered to be a unique integrated platform for subtraction enrichment (SE) and immunostaining-fluorescence in situ hybridization (iFISH) for the efficient detection of rare circulating cells, including CTCs shed from various solid epithelial tumors, circulating endothelial cells (CECs), and stem cells, represents this method [21]. Various methods for CTC detection have their own advantages and disadvantages. Therefore, for a long time, the methodology for the isolation and enrichment of CTCs has been under development.

### Clinical applications of CTC detection in HCC patients

The analysis of the specificity of identifying circulating HCC cells by detecting hepatocyte-or HCC-associated AFP mRNA in peripheral blood was first reported by Matsumura M et al. in 1994 [22]. The authors demonstrated that the level of AFP mRNA in blood was significantly increased in association with tumor size and serum AFP concentration. Extrahepatic metastasis was observed only in patients who had AFP mRNA in peripheral blood. In summary, the authors concluded that the presence of AFP mRNA in peripheral blood may be a useful marker of circulating malignant hepatocytes, which might be used to predict the hematogenous metastatic spread of tumor cells in patients with HCC. However, in that study, the authors did not find a significant correlation between elevated AFP levels and CTCs. In addition, a subsequent study by Matsumura et al. [23] reported a conclusion regarding the detection of AFP mRNA in CTCs in HCC using an RT-PCR method. They believed that the presence of AFP mRNA in blood is a predictor of outcomes in patients with HCC. However, other researchers reached a contradictory conclusion that although AFP mRNA can be used for the detection of circulating micrometastatic tumor foci in HCC, AFP mRNA in peripheral blood is not a specific marker of circulating micrometastases from HCC, especially in the context of surgical treatment of HCC [24]. Following these studies, the clinical utility of peripheral AFP mRNA was also explored at multiple research centers, and the controversy regarding its significance as prognostic marker persisted [25–30]. Hence, other tumor specific molecules in peripheral blood including MAGE-1, MAGE-3 [31], hTERT [29], GPC-3 [32], CD133, CD90 [33], K19, CD44 [34] and PLAC1 [35] have been investigated using RT-PCR to explore a direct correlation between the number of circulating CTCs and postoperative HCC recurrence. Although the results of several studies at present indicate that multiple HCC associated genes may be useful as clinical biomarkers for the early detection of cance, the evaluation of metastasis, the prediction of prognosis and the monitoring of treatment response, there is no widely recognized indicator yet. The problem is probably related to the fact that these markers are not specific to HCC. Moreover, RT-PCR based assays cannot accurately quantify the number of CTCs and are not able to provide intact CTCs for further research. It is thus imperative to establish other sensitive and specific methods for CTC enrichment and detection in HCC patients.

Based on a different principle than the RT-PCR method, the ISET technology, which provides a morphological, immunocytological and genetic characterization of individual CTCs, is widely used in CTC detection. The first application of the ISET method to detect CTCs in HCC patients was reported by Vona et al. in 2000 [36]. The authors considered the ISET technique to provide a unique opportunity for the cytological analysis of peripheral blood in oncology and for combining immunomorphological studies with novel assays to explore genetic abnormalities in individual isolated cells [37]. The CanPatrol CTC analysis platform (SurExam, China) is another enrichment technique for CTC isolation and characterization [38, 39]. This technique includes two major steps: a filter-based method to isolate CTCs and subsequent characterization of the CTCs using EMT markers, including the epithelial markers EpCAM and CK and the mesenchymal markers vimentin and twist.

Sun YF et al. [40] first described the possibility of detecting EpCAM-positive CTCs with the CellSearch™ system (CSS) in patients with HCC. The researchers tested blood samples from 123 HCC patients prior to resection and 1 month thereafter and detected ≥1 EpCAM(+) CTCs in 82 of the samples, among which 51 had ≥2 EpCAM(+) CTCs. They believed that a preoperative CTC count of ≥2 is a novel predictor for tumor recurrence in HCC patients after surgery, especially in patient subgroups with AFP levels of ≤400 ng/ml. Similarly, Schulze K et al. [41] detected ≥1 CTCs in 18/59 HCC patients and found that OS was significantly shorter in the CTC-positive cohort than in the CTC-negative cohort and therefore suggested that EpCAM-positive CTCs are frequently detectable in patients with advanced HCC and exhibit prognostic value in terms of OS and vascular invasion. A similar study by Kelley RK et al. reported ≥2 EpCAM(+) CTCs in 7/20 patients, showing a strong correlation between EpCAM(+) CTCs and AFP levels and vascular invasion [42]. With the continuous involvement of multiple research centers, these studies indicate that EpCAM(+) CTCs contribute to HCC recurrence and may therefore be used as a novel prognostic predictor for HCC patients. However, only a small proportion of HCC cells express EpCAM, which only identifies low numbers of CTCs in approximately 30–40% of patients [43]. Additionally, EMT, which is considered an initiation process for cancer metastasis, involves the loss of epithelial markers such as EpCAM, which means that the CSS may overlook circulating HCC cells. These reasons limit the continued use of the CSS in HCC patients.

Yin ZF et al. [44–46] used flow cytometry to identify circulating HCC cells using biomarkers such as Hep Par 1, CK and CPS1 and demonstrated that the unique magnetic circulating HCC cell separation system mediated by the interaction of the asialoglycoprotein receptor (ASGPR) with its ligand could be used for the specific and efficient detection of circulating HCC cells. In the study by Liu ZX et al. [47], CTCs in blood samples were analyzed by imaging flow cytometry based on the karyoplasmic ratio as well as EpCAM and CD 45. The authors found a strong association between CTC counts and the karyoplasmic ratio, presence of microvascular invasion (MVI), and HCC prognosis. With technological advancements, multimarker combinations, including pERK and pAkt [48], EMT markers (twist and vimentin) [49], MAGE-3 and survivin [50], CK, EpCAM and Glypican-3 [51], Annexin V, EpCAM, ASGPR1 and taMPs [52], were used in CTC detection for evaluating metastasis and prognosis and for monitoring the efficacy of sorafenib. In addition to the various detection methods mentioned above, the CTC-Chip has been considered an effective microfluidic device for capturing these EpCAM-expressing cells based on antibody-coated microposts [53–56]. Despite the diversity of detection methods and the importance of multiple molecular targets, multicenter trials are still needed to substantiate the claim that CTC detection will contribute to the future clinical management of HCC patients (Table 1).

**Table 1:** Circulating Tumor Cells Research In Hepatocellular Carcinoma

Ref.	Patients (HCC)	Ethnicity	Blood Source	Controls	Measurements	Methodology	Positive Rate
Matsumura M et al., 1994 [22]	33	Japan	Hepatic Vein Inferior Vena Cava	HV (30)	AFP	RT-PCR	52%
Funaki N et al., 1995 [103]	12	Japan	Peripheral blood	HV (5)	AFP	RT-PCR	71%
Lemoine A et al., 1997 [24]	20	France	Pre, During Peripheral blood	44 (IHC:2; LM: 25; LC: 13; HV: 2)	AFP	RT-PCR	17%
Matsumura M et al., 1999 [23]	88	Japan	Peripheral blood	NA	AFP	RT-PCR	63%
Wong IH et al., 2001 [25]	84	China	Pre, During, Post Peripheral blood	HV (53)	AFP ALB	RT-PCR	55%
Mou et al., 2002 [31]	30	China	Pre resection	HV (25)	MAGE1 MAGE3	RT-PCR	43%
Witzigmann et al., 2002 [26]	85	Germany	Pre, During, Post Peripheral blood	116 (OLT: 50; BLD: 39; HV: 27)	AFP	RT-PCR	28%
Cillo et al., 2004 [27]	50	Italy	Pre Resection Peripheral blood	50 (HD: 6; OCD: 44)	AFP	RT-PCR	40%
Jeng et al., 2004 [28]	81	China	Pre, Post Resection Peripheral blood	50 (HV: 30; HD: 69)	AFP	RT-PCR	23%
Kong et al., 2009 [29]	343	South Korea	Peripheral blood	NA	AFP hTERT	RT-PCR	59% 14%
Bahnassy AA et al., 2014 [33]	120	Egypt	Peripheral blood	63 (CLD: 30; HV: 33)	MAGE1/3 CK19 CD133	RT-PCR	55% 73% 69%
Choi GH et al., 2015 [34]	81	South Korea	Peripheral blood	16 (LHD)	K19, CD44	RT-PCT	22%
Jin JH et al., 2016 [30]	72	China	Peripheral blood	NA	AFP	RT-PCR	59%
Guo LM et al., 2017 [35]	51	China	Peripheral blood	30 (LC: 10; HV: 20)	CTAs PLAC1	RT-PCR	70%
Vona G et al., 2000 [36]	7	France	Peripheral blood	HV (8)	AFP	ISET RT-PCR	42%
Vona G et al., 2004 [104]	44	France	Peripheral blood	107 (HV:38; HD: 20)	NA	ISET	52%
Morris KL et al., 2014 [37]	52	United Kingdom	Peripheral blood	No treatment	GPC3	ISET CellSearch™	100%
Fan J et al., 2011 [105]	82	China	Pre, Post resection	NA	NA	CellSearch™	68%
Xu W et al., 2011 [44]	85	China	Pre resection	71 (HD: 37; HV: 20; OCD: 14)	Hep Par 1	CellSearch™	81%
Schulze K et al., 2013	59	Germany	Pre resection	HD (19)	AFP	CellSearch™	30%
Sun YF et al., 2013 [41]	123	China	Pre, Post resection	NA	CD133 ABCG2	CellSearch™	66%
Mu H et al., 2014 [106]	62	China	Peripheral blood	22 (CLD: 7; HV: 15)	ASGPR GPC3	CellSearch™	90% 93%
Guo W et al., 2014 [107]	299	China	Peripheral blood	120 (HV: 71; BT: 24; CLD: 25)	EpCAM	CellSearch™	76%
Kelley RK et al., 2015 [42]	20	Caucasian 55% Asian 35% American 10%	Peripheral blood	NA	NA	CellSearch™	40%
Zhou Y et al., 2016 [108]	49	China	Peripheral blood	HV (50)	CD4	CellSearch™	35%
Felden JV et al., 2017 [109]	61	Germany	Peripheral blood	CLD (31)	NA	CellSearch™	15%
Sun YF et al., 2017 [40]	73	China	Peripheral blood Portal Vein	NA	EpCAM	CellSearch™	68%
Xu Wen et al., 2011 [44]	85	China	Peripheral blood	37 (CLD)	Her-2 TP53	Flow Cytometry	81%
Li YM et al., 2013 [49]	60	China	Peripheral blood	30 (BT: 10; HV: 10; OLT: 10)	Twist Vimentin	Flow Cytometry	76%
Li J et al., 2014 [45]	27	China	Peripheral blood	61 (OLT: 12; CLD:23; BT: 11; HV: 15)	ASGPR CPS1 P-CK	Flow Cytometry	89%
Liu HY et al., 2015 [46]	32	China	Peripheral blood	77 (OLT: 17; CLD: 40; HV: 20)	ASGPR CPS1	Flow Cytometry	91%
Liu ZX et al., 2016 [47]	52	China	Peripheral blood	HV (12)	EpCAM	Flow Cytometry	57%
Li J et al., 2016 [48]	109	China	Peripheral blood	NA	pERK pAkt	Flow Cytometry	92%
Shi J et al., 2016 [50]	47	China	Pre, Post Resection Peripheral blood	NA	EpCAM	Flow Cytometry	100%
Ogle LF et al., 2016 [51]	69	United Kingdom	Peripheral blood	31 (HV: 15; CLD: 16)	Cytokeratin EpCAM	Flow Cytometry	29% 18%
Julich-Haertel H et al., 2017 [52]	172	Germany	Peripheral blood	256 (CLD: 54; HV: 202)	EpCAM ASGPR1 taMRs	Flow Cytometry	78%
Zhang Yu et al., 2016 [55]	36	China	Peripheral blood	NA	CPS1 CD45	CTC-Chip	85%
Kalinich M et al., 2016 [54]	16	United States	Peripheral blood	NA	Chip	CTC-Chip	56%
Wang S et al., 2016 [56]	42	China	Peripheral blood	NA	Chip	CTC-Chip	61%
Wang Z et al., 2017 [38]	62	China	Peripheral blood	NA	NA	CanPatroI™ System	84%
Chen J et al., 2017 [39]	195	China	Peripheral blood	NA	NA	CanPatroI™ System	95%

*HV* Healthy Volunteers, *IHC* Cholangiocarcinoma, *LM* Liver Metastases, *LC* Liver Cirrhosis, *NA* Not Applicable, *OLT* Other malignant liver tumors, *HD* Health disease without evidence of HCC, *OCD* Other cancerous disease, *CLD* Chronic Liver Disease, *LHD* Liver healthy donors, *BT* Benigh tumor

### Future directions

CTC analysis might provide personalized and effective strategies for clinicians and researchers because CTC are sensitive biomarkers that enable early diagnosis, real-time monitoring, and molecular characterization to facilitate the implementation of precision medicine. In a meta-analysis reported by Sun C et al., they demonstrated that CTC assay is not recommended as an independent HCC diagnostic tool, but is associated with poor clinicopathologic characteristics of HCC patients and could indicate poor prognosis. In addition, they systematically synthesized diverse study results and provide powerful evidence for the potential clinical value of CTC assay [57]. However, numerous bottlenecks must be overcomed before CTC analysis can be applied in the clinic. One of the challenges is the inconsistency among detection methods. The various methods of CTC detection mentioned above have their own advantages and disadvantages. It is extremely challenging to establish a highly sensitive and specific method that can capture the full spectrum of CTCs. Therefore, standardized assay protocols for CTC analysis, including sample preparation, enrichment and detection, are critical. In addition, most studies are single-center case-control research, with limited sample size. Validation is sometimes difficult if not completely nonexistent. There is a need for a multicenter prospective studies with a sufficient sample size and long follow-up to evaluate CTC detection methodologies. In multicenter studies, the detection method is uniform, and large samples can provide powerful validation for accurate analysis and standard evaluation of the final data. Although CTC detection is currently only performed for research, ongoing advancements in technology will make it feasible in clinical practice in the near future.

### Biology, detection and enrichment of ctDNA

Before introducing ctDNA, we need to introduce the concept of circulating cell-free nucleic acids (cfNAs) comprising DNA, mRNA and miRNAs that were discovered in human peripheral blood samples [58]. The first report of cfNAs in human peripheral blood was published in 1948 by Mandel and Metais [13]. However, their work did not gain enough attention until thirty years later with the discovery of higher concentrations of cell-free DNA (cfDNA) in serum and plasma from cancer patients than in those from healthy individuals [59]. Currently, cfDNA is considered to be secreted into peripheral blood in the physiological state by normal cells at an average concentration of 30 ng/ml (0–100 ng/ml) [60]. ctDNA represents tumor-derived fragmented DNA in the bloodstream of cancer patients with a constitution that varies substantially from < 0.01% to > 60% of alleles in circulation [61, 62]. ctDNA carries the genetic information of the tumor, and quantitative or qualitative analysis of ctDNA has important clinical value for early diagnosis, treatment, and progression monitoring of tumors. The concentration of cfDNA were accompanied by a decrease in DNase activity because cfDNA is degraded by peripheral blood deoxyribonuclease activity. The normal cells in peripheral circulation can also release cfDNA, and this reduces ctDNA concentrations [63]. For ctDNA to be used as a liquid biopsy tool, the key is to be able to distinguish ctDNA from the large amount of cfDNA using existing advanced technology.

At present, there is a debate about the collection and extraction methods for retrieving ctDNA from serum or plasma. cfDNA lysis occurs secondary to the clotting process of blood cells in collection tubes; thus, several studies have found significantly high cfDNA concentrations in serum than in plasma [64, 65]. As we mentioned earlier, this further reduces the concentration of ctDNA. Similarly, improper specimen collection or mechanical processing of blood leads to the destruction of the blood cells, causing the release of cfDNA into plasma [66]. Until recently, the mainstream view of many researchers indicated a preference of cfDNA analysis in the plasma fraction over that in serum [67]. Although plasma is theoretically less likely to be contaminated with DNA from blood cells, the amount of DNA in plasma is more or less affected due to the time interval between blood collection and analysis [66]. Three different tubes are recommended for the collection of blood. EDTA tubes are usually the first recommended collection tube if the blood is to be processed within 6 h, but if the blood needs be stored for a longer period of time (> 6 h) before being processed, Streck or CellSave blood collection tubes (Omaha, NE, USA) may be better options [68]. These details are key in the process of ctDNA extraction and directly affect the stability and accuracy of the process.

### Detection methods for ctDNA

Generally, methods for the detection of ctDNA should be highly sensitive and specific because 1 ml of blood can be used to extract 10 ng of cell free DNA, of which only 1% or even 0.01% of the overall circulating tumor DNA [69]. Based on differences in testing purposes, the detection methods for ctDNA may also be different. The detection methods may be summarized as follows: targeted methods to assay a few known mutations using PCR (e.g., digital PCR, BEAMing (beads, emulsion, amplification, and magnetics) digital PCR, amplification-refractory mutation system (ARMS)-PCR) and untargeted methods to sequence millions of DNA fragments (e.g., Sanger sequencing, next-generation sequencing (NGS)). According to the different enrichment strategies, NGS-based technologies can be divided into targeted amplification sequencing (TAS) and targeted capture sequencing (TCS). TAS involves the use of dozens or even hundreds of pairs of PCR primers for the target gene for multiple rounds of PCR amplification and enrichment, and a representative method is tagged-amplicon deep sequencing (TAM-Seq) [70]. TCS involves the use of a probe for capturing the targeted gene by using hybrid method of enrichment. The most classic TCS method is cancer personalized profiling by deep sequencing (CAPP-Seq) [71].

### Clinical application of ctDNA detection in HCC patients

ctDNA carries information on tumor-specific genetic or epigenetic alterations, such as point mutations, copy number variations (CNVs), chromosomal rearrangements, and DNA methylation patterns and offers a unique opportunity for serially monitoring tumor genomes in a noninvasive, convenient and accurate manner. Two different changes are monitored during the detection of ctDNA: quantitative changes and qualitative changes. The first detection method measures the quantity of ctDNA in circulation, and the second detects tumor-specific genetic aberrations. Many studies have investigated quantitative changes in cfDNA in the blood of HCC patients and demonstrated that elevated levels of cfDNA may represent a novel complementary tool with potential clinical applications for screening, detection, treatment monitoring and predicting metastatic potential in HCC [72–78]. For example, Ren N et al. [72] demonstrated that the combination of circulating plasma DNA level and allelic imbalance (AI) at D8S258 might be an independent predictor for the prognosis of HCC. Circulating plasma DNA level were detected in 79 HCC patients, and AI at D8S258 was significantly correlated with tumor differentiation, TNM stage and vascular invasion and negatively correlated with the 3-year disease-free survival (DFS) and OS. GSTP1 cfDNA levels were found to be significantly increased in the sera of patients with HCV-associated HCC in the study by Lizuka N et al. [73]. They believed that circulating GSTP1 cfDNA is a good and specific biomarker for HCV-associated HCC. Similarly, Yan L et al. [78] analyzed an HCC index including age, cfDNA and AFP for the diagnosis of HCC with 87% sensitivity and 100% specificity. DNA methylation is one of the earliest known modification pathways, and a large number of studies have shown that DNA methylation can lead to changes in chromatin structure, DNA conformation, DNA stability and DNA and protein interactions, thus controlling gene expression. Several studies have revealed that alterations in DNA methylation at many genes, including *p15* [79], *p16* [80], *APC* [81], *SPINT2* [82], *SFRP1* [83], *p16INK4a* [84], *TFPI2* [85], *GSTP1* [86] and *RASSF1A* [87, 88], are associated with the initiation and progression of HCC. For example, the Ras association domain family protein 1A (RASSF1A) is a tumor suppressor that is frequently lost in human cancers by promoter-specific methylation. Mohamed, N. A et al. [89] showed that RASSF1A gene hypermethylation could be detected in the serum of 90% of HCC patients and 62.5% of HCV patients, while only 10% of healthy volunteers displayed hypermethylation at this gene. Logistic regression analysis further identified that serum levels of methylated RASSF1A could be used to differentiate HCC patients from healthy volunteers, with an area under the receiver operating characteristics curve (AUROC) of 0.83 nmol/l and overall predictive accuracy of 77.5%. Taken together, these findings indicate that serum levels of methylated RASSF1A may be useful for the early diagnosis of HCC, especially in high-risk patients with HCV infection. The detection of methylation in peripheral DNA has great potential for diagnostic, prognostic, and therapeutic efficacy evaluations in HCC, but the most important aspect is its diagnostic value. A large number of hypermethylated genes, such as *DBX2* [90], *TGR5* [91], *MT1M*, *MT1G* [92] and *INK4A* [93], in cfDNA from HCC patients were identified as biomarkers or vascular invasion. Although a high degree of methylation at multiple genes has been shown to play an important role in the process of HCC diagnosis, there is no recognized indicator confirmed in multiple centers. In addition, the combined detection of the methylation status of multiple genes may be an effective way to improve the diagnostic efficiency [94]. To evaluate the potential of ctDNA methylation markers for diagnosing and evaluating the prognosis of HCC, Xu RH et al. [94] compared differential methylation profiles of HCC tissues and blood leukocytes in healthy individuals and identified a methylation marker panel that is enriched in HCC. The sensitivity and specificity of this diagnostic prediction model with ten markers in a training data set of 715 HCC samples and 560 normal samples were 85.7% and 94.3%, respectively. Using UniCox and LASSO-Cox methods, an 8-marker panel was constructed to predict the prognosis of HCC patients. A combined prognostic score (cp-score) with these markers was significantly correlated with the risk of death both in the training and validation data set, and the cp-score was an independent risk factor for survival. In addition to methylation-based assays of ctDNA, genetic alterations such as mutations, deletions, epigenetic changes can also be used as tumor biomarkers in HCC. Until recently, many studies have confirmed that tumor-specific mutations in TP53 [95], ITH [96], HCK [97], CTNNB1 and TERT [98] are common in the peripheral blood of patients with HCC. Jiang P et al. [99] applied the established CAZA mathematical model to calculate CNVs in tumors by sequencing DNA. Furthermore, abnormal CNVs in two patients with hepatitis B were found using this model, and the occurrence of HCC was also observed during the follow-up. Therefore, cfDNA has potential clinical utility as a biomarker for the early diagnosis of HCC and for the prediction of drug resistance and prognostic outcomes in patients with HCC (Table 2).

**Table 2:** Circulating cfDNA In Hepatocellular Carcinoma

Ref.	Patients (HCC)	Ethnicity	Blood sample	Controls	Measure methods	Target site	Positive rate
Ren N et al., 2006 [71]	79	China	Plasma	40 (LC: 20; HV: 20)	Quantitative analysis Real-Time PCR	Chromosome 8p	NA
Iizuka N et al., 2006 [72]	60	Japan	Serum	CLD: 30	Quantitative analysis Real-Time PCR	GSTP1	NA
Yang Y et al., 2011 [73]	60	China	Plasma	50 (CLD: 21; HV: 29)	Quantitative analysis FQ-PCR	hTERT	NA
Huang Z et al., 2012 [74]	72	China	Plasma	78 (LC: 72; HV: 41)	Quantitative analysis Real-Time PCR	Beta-Actin	NA
Piciocchi M et al., 2013 [75]	66	Italy	Plasma	76 (LC: 35; CLD: 41)	Quantitative analysis Real-Time PCR	hTERT	NA
Huang A et al., 2016 [76]	53	China	Plasma	16: OLT	Quantitative analysis Real-Time PCR	ALU	NA
Yan L et al., 2017 [77]	24	China	Plasma	62: CLD	Quantitative analysis Qubit Assay	NA	NA
Wong, I. H et al., 2000	25	China	Plasma /Serum	55 (LC: 35; HV: 20)	Methylation MS-PCR	*P15* *P16*	64% 48%
Wong, I. H et al., 2003	29	China	Plasma	CLD: 50	Methylation MS-PCR	*P16INK4a*	80%
Yeo, W et al., 2005 [86]	40	United Kingdom	Plasma	HV: 10	Methylation MS-PCR	*RASSF1A*	92%
Zhang YJ et al., 2007 [79]	50	China	Plasma	HV: 50	Methylation MS-PCR	*RASSF1A* *P15* *P16*	70% 22% 44%
Chang Hong et al., 2008 [85]	26	China	Plasma	CLD: 16	Methylation MS-PCR	P16 GSTP1 RASSF1A APC	19% 19% 26% 61%
Chan, K. C et al., 2008 [87]	63	China	Serum	HV: 50	Methylation MS-PCR	RASSF1A	93%
Iyer, P et al., 2009	150	Egypt	Plasma	HV: 150	Methylation MS-PCR	APC FHIT P15 P16	53% 67% 10% 46%
Huang, Z. H et al., 2011 [82]	72	China	Plasma	HD: 37	Methylation MS-PCR	APC GSTP1 RASSF1A SFRP1	68% 55% 72% 55%
Iizuka, N et al., 2011 [81]	108	Japan	Serum	CLD: 56	Methylation MS-PCR	BASP1 CCND2 APC SPINT2 SRD5A2 CFTR RASSF1A	62% 64% 17% 35% 8% 56% 92%
Sun, F. K et al., 2012 [84]	43	China	Serum	50 (CLD: 24; HV: 26)	Methylation MS-PCR	TFPI2	46%
Zhang, P et al., 2013 [89]	31	China	Serum	HV: 21	Methylation MS-PCR	DBX2 THY1	88% 85%
Han, LY et al., 2014 [90]	160	China	Serum	133 (CLD: 88; HV: 45)	Methylation MS-PCR	TGR5	48%
Ji, XF et al., 2014 [91]	121	China	Serum	69 (CLD: 37; HV: 31)	Methylation MS-PCR	MT1M MT1G	48% 70%
Huang, G et al., 2014 [92]	66	United States	Serum	CLD:43	Methylation MS-PCR	INK4A	65%
Wen Lu et al., 2015	36	China	Serum	55 (LC: 17; HV: 38)	Methylation MS-PCR	RGS10 ST8SIA6 RUNX2 VIM	94%
Xu RH et al., 2017 [93]	1098	China	Plasma	HV: 835	Methylation MS-PCR	Diagnostic panel (10) Prognostic panel (8)	85%

*HV* Healthy Volunteers, *IHC* Cholangiocarcinoma, *LM* Liver Metastases, *LC* Liver Cirrhosis, *NA* Not Applicable, *OLT* Other malignant liver tumors, *HD* Health disease without evidence of HCC, *OCD* Other cancerous disease, *CLD* Chronic Liver Disease, *LHD* Liver healthy donors, *BT* Benigh tumor

### Future directions

Profiling the molecular changes in tumors is important for guiding appropriate targeted therapy. In addition to the guidance of molecular targeted treatment, ctDNA detection could potentially help to monitor treatment response as the mutational status in plasma has been demonstrated to reflect the tumor burden in patients and to be correlated with the clinical status of patients [100]. In subsequent studies in HCC, research strategies for ctDNA analysis can be divided into two categories. First, cancer-associated changes including point mutations/indels, DNA methylation or chromosomal aberrations, can be identified by the analysis of tumor tissues, followed by the identification and quantification of corresponding tumor-specific changes in the plasma. This strategy may provide powerful data for subsequent targeted therapies in HCC patients. Second, ctDNA detection in plasma can be used for direct screening of cancer-associated changes, and for screening or surveillance of HCC. Simultaneously, changes in ctDNA can also provide a basis for the timing of transarterial chemoembolization (TACE) in patients with HCC.

The tyrosine kinase inhibitor (TKI) sorafenib has been the standard systemic treatment option in patients with locally advanced HCC for several years. In resent years, many trials have been performed to investigate the use of other TKIs in first- or second- line treatment; however, only regorafenib, cabozantinib and lenvatinib showed sufficient efficacy and reached their primary end points in their respective phase 3 trials [101]. Immunotherapy has also finally entered the stage for the treatment of HCC [102]. Nevertheless, the prediction of response to immunotherapy or TKIs has not been reliable in HCC. Tumor mutational burden may be a potential predictor for response to these treatment. In addition, the quantification of mutations in HCC using ctDNA has been shown to be a very good predictor for response to immunotherapy and TKIs. Thus, there is an urgent need to evaluate and develop the use of ctDNA to check if it can be a good tool to assess responses to immunotherapy or TKI.

## Conclusion

The main advantage of liquid biopsy analysis is the unique potential of CTCs and ctDNA to be conveniently obtained through minimally invasive methods at multiple time points over the course of disease. Further research on the molecular characterization of ctDNA and CTCs will provide a better understanding of the development of resistance to sorafenib or TACE and help establish more personalized treatment plans with lower cost and fewer side effects for HCC patients. These data may have a profound impact on the use of this particular strategy for patients and may play a role in the selection of patients receiving treatment. Liquid biopsy has made it possible to screen for HCC in the early stages and has shown promise in the areas of tumor diagnostics, treatment and monitoring. Additionally, the benefits of liquid biopsy make it a promising tool for monitoring the development of tumors, with extremely high clinical application value and market prospect.

Because of the differences in experimental design and detection methods for CTCs and ctDNA accross studies, the experimental data are very diverse and unreliable. The standardization of detection methods and the precision of detecting biomarkers are key factors for the application of CTCs and ctDNA. With the accumulation liquid biopsy data, along with the biology and complexity of HCC, the presence of only a few indicators for the distinction between tumors and non-tumor patterns will lead to a shift to research models based on big data and artificial intelligence. Therefore, mutations at multiple loci, detection panels involving multiple methylation patterns and multiple immune biomarkers using CTC and ctDNA will be used for therapeutic monitoring, prognostic evaluation and risk assessment in HCC.

## Core tips

Hepatocellular carcinoma is a leading cause of cancer death worldwide. As CTCs and ctDNA in HCC patients harbor the molecular characteristics of HCC cells, liquid biopsy analysis in blood may be sufficient for providing convenient, noninvasive and accurate information for HCC diagnosis, treatment and prognostic evaluations. In this review, we will summarize and discuss current research progress and challenges in the application of liquid biopsy in HCC.

## Abbreviations

cfNAs: Cell-free nucleic acids
CTC: Circulating tumor cells
ctDNA: Circulating tumor DNA
EMT: Epithelial transmutation
EpCAM: Epithelial cell adhesion molecule
HBV: Hepatitis B virus
HCC: Hepatocellular carcinoma
HCV: Hepatitis C virus
TACE: Transarterial chemoembolization
TKI: Tyrosine-kinase inhibitors
